# Occult Nodal Disease in Gallbladder Cancer: An International Multi-institutional Analysis and Preoperative Risk Stratification

**DOI:** 10.1245/s10434-026-19460-0

**Published:** 2026-03-17

**Authors:** Jun Kawashima, Kizuki Yuza, Yutaka Endo, Kota Sahara, Federico Aucejo, Hugo P. Marques, Tom Hugh, Minoru Kitago, Andrea Ruzzenente, Yuki Homma, Itaru Endo, Timothy M. Pawlik

**Affiliations:** 1 Department of Gastroenterological Surgery, Yokohama City University, Yokohama, Japan; 2 Department of Surgery, The Ohio State University Wexner Medical Center and James Comprehensive Cancer Center, Columbus, OH USA; 3 Department of Transplant Surgery, University of Rochester Medical Center, Rochester, NY USA; 4 Department of General Surgery, Cleveland Clinic, Digestive Disease and Surgery Institute, Cleveland, OH USA; 5 Department of Surgery, Curry Cabral Hospital, Lisbon, Portugal; 6 Department of Surgery, The University of Sydney, Sydney, NSW Australia; 7 Department of Surgery, Keio University, Tokyo, Japan; 8 Division of General and Hepatobiliary Surgery, University of Verona, Verona, Italy

## Abstract

**Introduction:**

Accurate preoperative nodal staging remains challenging in gallbladder cancer (GBC), and a substantial proportion of patients presumed to be clinically node-negative have nodal metastasis at surgery. This study aimed to quantify the burden of occult nodal disease (OND)—defined as pathologic node-positive disease among clinically node-negative patients—and to identify preoperative factors associated with OND.

**Methods:**

Patients who underwent upfront curative-intent resection with regional lymphadenectomy for GBC were identified from an international multi-institutional database. Among patients staged as clinically node-negative (cN0) on preoperative imaging, multivariable logistic regression was used to identify preoperative predictors of OND. CA19-9 and the systemic immune-inflammation index (SII) were log-transformed for modeling purposes.

**Results:**

Among 187 patients, 142 (75.9%) were classified as cN0 preoperatively, among whom 47 (33.1%) had OND on final pathology. On multivariable analysis, higher ln(SII) (odds ratio [OR] 1.69, 95% confidence interval [CI] 1.05–2.86), higher ln(CA19-9) (OR 1.30, 95% CI 1.12–1.53), and preoperative jaundice (OR 3.68, 95% CI 1.21–11.76) were independently associated with OND. The observed OND rate increased stepwise with the number of elevated preoperative markers (SII > 890.2, CA19-9 > 37 U/mL, and jaundice): 17.1% with 0 markers, 44.8% with 1 marker, and 73.6% with 2–3 markers.

**Conclusions:**

OND was present in approximately one-third of clinically node-negative GBC patients undergoing lymphadenectomy. Preoperative jaundice, elevated CA19-9, and elevated SII independently predicted OND and provided simple risk stratification. Incorporating these readily available markers into preoperative assessment may improve risk enrichment for OND and help guide additional staging and treatment sequencing.

**Supplementary Information:**

The online version contains supplementary material available at 10.1245/s10434-026-19460-0.

Gallbladder cancer (GBC) is the most common malignancy within biliary tract cancers (BTCs).^1,2^ Curative-intent resection remains the only potentially curative treatment option; however, many patients present with advanced disease, and fewer than one in four symptomatic patients are amenable to surgical resection.^3,4^ Even among patients who undergo curative-intent resection, long-term outcomes are heterogeneous and largely determined by disease extent.^5^ Indeed, 5-year overall survival (OS) ranges from 12% to 95% across American Joint Committee on Cancer (AJCC) T and N categories.^6^ Together, these findings emphasize the need for accurate preoperative staging and for treatment strategies that go beyond surgery alone to optimize outcomes for patients with biologically or anatomically advanced disease.

Among prognostic factors, lymph node metastasis (LNM) is consistently one of the strongest predictors of poor survival in GBC, and outcomes remain dismal once nodal involvement is present.^5,7,8^ Because nodal disease may reflect an increased risk of systemic dissemination, patients with clinically apparent LNM may be more appropriately managed with neoadjuvant systemic therapy rather than upfront surgery in selected settings. In line with this concept, recent international consensus efforts have characterized node-positive disease as borderline resectable and have recommended consideration of preoperative chemotherapy for appropriate candidates.^9^ Therefore, accurate preoperative assessment of nodal status is important to guide multidisciplinary treatment selection.

Occult nodal disease (OND)—pathologically confirmed nodal metastasis in patients without clinical or radiographic evidence of nodal involvement—has been increasingly recognized across multiple malignancies and is associated with important prognostic and therapeutic implications.^10–13^ In GBC, however, OND remains insufficiently characterized. Contemporary imaging modalities, including computed tomography (CT), magnetic resonance imaging (MRI), and positron emission tomography (PET), have limited sensitivity to detect nodal metastasis, resulting in a subset of patients undergoing upfront surgery under the assumption of node-negative disease but ultimately being found to harbor nodal metastasis on surgical pathology.^14–16^ This scenario may represent a missed opportunity for improved preoperative risk stratification and treatment optimization. Accordingly, identifying clinically applicable preoperative markers that enrich for OND could improve staging accuracy and inform individualized management strategies. In this context, the present study used a large, international multi-institutional cohort to characterize the prevalence of OND among patients who underwent curative-intent surgery for GBC, as well as identify preoperative factors associated with OND to support improved risk stratification in GBC.

## Methods

### Data Source and Patient Selection

Patients who underwent upfront curative-intent resection with lymphadenectomy for GBC between 2008 and 2022 were identified from an international multi-institutional database including data from major Western and Eastern hepatobiliary centers. Patients were excluded if data on preoperative lymph node (LN) assessment were missing. In addition, patients who received preoperative systemic therapy were excluded to focus on an upfront-surgery cohort. Preoperative nodal status was determined based on preoperative cross-sectional imaging (CT and/or MRI) with PET/CT used when available according to each institution’s standard practice. Clinically metastatic LNs were defined by imaging features consistent with metastasis, including 1) short-axis diameter ≥10 mm; 2) short-axis diameter <10 mm but suspicious morphology and/or enhancement pattern similar to the primary tumor; 3) evidence of extranodal extension (e.g., perinodal fat stranding); or 4) increased uptake on PET-CT.^17^ The study was approved by the institutional review boards of all participating institutions.

### *Variables and Outcomes*

Patient demographic and clinicopathologic variables included age, sex, American Society of Anesthesiologist (ASA) classification, geographic region (i.e., Western countries, Eastern countries), presence of incidental diagnosis, preoperative cholecystitis, preoperative jaundice, preoperative carbohydrate antigen 19-9 (CA19-9), preoperative systemic immune-inflammation index (SII), preoperative LN status (i.e., cN0, cN+), use of PET-CT for preoperative LN assessment, type of surgery (i.e., extended cholecystectomy, cholecystectomy + S4b/5 resection, cholecystectomy + other liver resection), the total number of LNs examined (TLNE), pathological T category based on the AJCC 8th edition,^18^ pathological N category based on AJCC 8th edition, surgical margin (i.e., R0, R1), microvascular invasion (MVI), tumor grade (i.e., well, moderate, poorly, undifferentiated), perineural invasion (PNI), postoperative severe complication, and receipt of adjuvant chemotherapy.

Preoperative jaundice was defined as a peak preoperative total bilirubin level exceeding 2.0 mg/dL.^19^ SII was calculated as the neutrophil-to-lymphocyte ratio multiplied by the platelet count.^20^ The prespecified cutoff value for SII (890.2) and CA19-9 (37 U/mL) was based on prior literature.^20,21^ Severity of postoperative complications was defined according to the Clavien-Dindo classification system (grade I-V); severe complications were defined as Clavien-Dindo classification ≥III.^22^

The primary outcome was OND, defined as pathologic LNM (pN+) among patients assessed as clinically node-negative (cN0) on preoperative imaging. The secondary outcome was the diagnostic performance of preoperative LN assessment for identifying pathologic nodal metastasis. Using final pathologic nodal status as the reference standard, sensitivity, specificity, positive predictive value, and negative predictive value of preoperative imaging-based nodal staging were calculated.

### Statistical Analysis

Descriptive statistics were presented as median values with interquartile ranges (IQR) for continuous variables and as frequencies with percentages for categorical variables. Continuous variables were compared by using the Mann-Whitney *U* or Kruskal-Wallis tests, as appropriate. Categorical variables were compared with the χ^2^ test or Fisher’s exact test. Multiple imputation by chained equations (MICE) was utilized to handle missing values.^23^ To assess the diagnostic performance of preoperative LN assessment, patients were categorized as clinically node-positive (cN+) or clinically node-negative (cN0) based on the preoperative criteria described above and were compared against pathologic nodal status (pN+ vs. pN0) to construct 2×2 contingency tables for performance estimates.

Among patients with clinically node-negative (cN0), logistic regression analysis was used to assess the association between various clinicopathologic factors and OND among patients who underwent LND. Several potential perioperative prognostic predictors were selected based on clinical importance. Variables significantly associated with OND in univariable analysis (*p* < 0.10) were subsequently included in the multivariable model. Because CA19-9 and SII were right-skewed, these variables were natural log–transformed for regression modeling (ln(CA19-9) and ln(SII)). To assess potential non-linear relationships for these continuous biomarkers, adjusted restricted cubic splines with three knots were fitted by using the rms package, and the adjusted predicted probability of OND was plotted across the observed ranges of ln(CA19-9) and ln(SII). To visualize the joint association between CA19-9 and SII, we generated contour plots of model-predicted probabilities across a grid of CA19-9 and SII values, stratified by the presence of preoperative jaundice. Model discrimination was assessed by the area under the receiver operating characteristic curve (AUC), and internal validation was performed by using bootstrap resampling to estimate optimism-corrected AUC. Statistical significance was set at α = 0.05. All analyses were performed using R version 4.4.2 (R Project for Statistical Computing, Vienna, Austria).

## Results

### Patient Demographics

Among 187 patients who met inclusion criteria, 75 (40.1%) were male, and the median age was 70 years (IQR 61–76). Overall, 90 (48.1%) patients had an ASA class greater than 2, and 36 (19.3%) presented with preoperative jaundice. The median preoperative CA19-9 level was 15.0 U/mL (IQR 7.0–119.0), and the median SII was 670.3 (IQR 441.8–1306.2); 70 (37.4%) patients were classified as having high SII (>890.2). With respect to operative management, 75 (40.1%) patients underwent extended cholecystectomy, whereas 98 (52.4%) underwent cholecystectomy with S4b/S5 resection. The median TLNE was 6 (IQR 3–9). The distribution of TLNE across participating institutions is summarized in Supplementary Fig. 1; the institution with the lowest TLNE had a median of 4 (IQR 2–6), whereas the institution with the highest TLNE had a median of 12 (IQR 8–15.5). Pathologically, 73 (39.1%) patients had T3 or T4 disease. Overall, 66 (35.3%) and 12 (6.4%) patients had pN1 and pN2 disease, respectively. R0 resection was achieved in 144 (77%) patients. MVI was identified in 107 (57.2%) patients; poorly differentiated or undifferentiated tumors and PNI were observed in 50 (26.7%) and 100 (53.5%) patients, respectively. Postoperatively, 29 (15.5%) patients experienced severe complications, and 91 (48.7%) received adjuvant chemotherapy (Table 1).

**Table 1:** Clinicopathological characteristics of the analytic cohort

Characteristics	All patients
	*n* = 187
Age, yr, median (IQR)	70 [61, 76]
Sex, male	75 (40.1)
ASA classification, > 2	90 (48.1)
Geographic region, western countries	152 (81.3)
Incidental diagnosis	95 (50.8)
Cholecystitis	44 (23.5)
Jaundice	36 (19.3)
CA19-9, median (IQR)	15.0 [7.0, 119.0]
≤ 37 U/mL	117 (62.6)
> 37 U/mL	70 (37.4)
SII, median (IQR)	670.3 [441.8, 1306.2]
≤ 890.2	117 (62.6)
> 890.2	70 (37.4)
Preoperative lymph node status, node positive	45 (24.1)
PET-CT for preoperative LN assessment	26 (13.9)
*Type of surgery*	
Extended cholecystectomy	75 (40.1)
Cholecystectomy + S4b-S5 resection	98 (52.4)
Cholecystectomy + other liver resection	14 (7.5)
TLNE, median (IQR)	6 [3, 9]
*Pathological T category*	
T1	23 (12.3)
T2	91 (48.7)
T3	59 (31.6)
T4	14 (7.5)
*Pathological N category*	
N0	109 (58.3)
N1	66 (35.3)
N2	12 (6.4)
Surgical margin, R0	144 (77.0)
Microvascular invasion	107 (57.2)
Grade, poor/undifferentiated	50 (26.7)
Perineural invasion	100 (53.5)
Severe complication	29 (15.5)
Adjuvant chemotherapy	91 (48.7)

Values are numbers with percentages in parentheses unless otherwise indicated

*ASA*, American Society of Anesthesiologists; *CA19***-***9*, carbohydrate antigen 19-9; *SII*, systemic immune-inflammation index; *PET-CT*, positron emission tomography-CT; *LN*, lymph node; *TLNE*, total number of lymph nodes examined; *IQR*, interquartile range

### Preoperative Nodal Assessment and Diagnostic Performance

Preoperatively, 142 (75.9%) patients were classified as clinically node-negative (cN0) and 45 (24.1%) as clinically node-positive (cN+). PET-CT was used for nodal assessment in 26 patients (13.9%). On final pathologic evaluation, 78 (41.7%) patients had LNM. When preoperative nodal status was compared with pathologic nodal status, 31 patients were true positives (cN+/pN+), 14 were false positives (cN+/pN0), 47 were false negatives (cN0/pN+), and 95 were true negatives (cN0/pN0). Accordingly, the sensitivity was 39.7%, specificity was 87.2%, positive predictive value was 68.9%, and negative predictive value was 66.9%.

Among the 26 patients who underwent PET-CT as part of preoperative nodal assessment, 22 (84.6%) patients were classified as cN0 and four (15.4%) as cN+. Comparing preoperative nodal status with pathologic nodal status in this subgroup, two patients were true positives (cN+/pN+), two were false positives (cN+/pN0), five were false negatives (cN0/pN+), and 17 were true negatives (cN0/pN0). Accordingly, the sensitivity was 28.6%, specificity was 89.5%, positive predictive value was 50%, and negative predictive value was 77.3%. In contrast, among patients who did not undergo PET-CT (n = 161), 120 (74.5%) were classified as cN0 and 41 (25.5%) as cN+. When preoperative nodal status was compared with pathologic nodal status in this subgroup, 29 patients were true positives (cN+/pN+), 12 were false positives (cN+/pN0), 42 were false negatives (cN0/pN+), and 78 were true negatives (cN0/pN0), yielding a sensitivity of 40.8%, specificity of 86.7%, positive predictive value of 70.7%, and negative predictive value of 65%.

### Risk Factors for OND Among Patients with Clinically Node-Negative

**Fig. 1:**
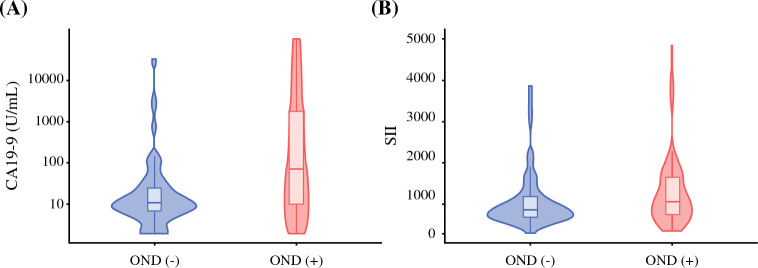
Distribution of preoperative carbohydrate antigen 19-9 (CA19-9) (**A**) and systemic immune-inflammation index (SII) (**B**) by occult nodal disease (OND) status

Among the 142 patients classified as clinically node-negative (cN0), OND was identified in 47 patients (33.1%). Compared with patients without OND, individuals with OND more frequently presented with preoperative jaundice (12 [25.5%] vs. 8 [8.4%], *p* = 0.012) and had higher preoperative CA19-9 levels (median, 70 U/mL [IQR 10–1,849] vs. 11 U/mL [IQR 7–25]; *p* < 0.001), as well as higher SII (median, 800.3 [IQR 483.7–1462.8] vs. 594.3 [IQR 426.1–950.6]; *p* = 0.03) (Fig. 1). In addition, the median TLNE was higher among patients with OND (7 [IQR 3–13] vs. 5 [IQR, 3–8]; *p* = 0.023). On pathologic evaluation, OND was more commonly observed in patients with T3/T4 disease (23 [49%] vs. 19 [20%]; *p* < 0.001) and was associated with a lower rate of R0 resection (31 [66%] vs. 89 [93.7%]; *p* < 0.001). Patients with OND also more frequently had microvascular invasion (34 [72.3%] vs. 41 [43.2%]; *p* = 0.002) and PNI (31 [66%] vs. 36 [37.9%]; *p* = 0.003). Postoperatively, adjuvant chemotherapy was administered more often to patients with OND (35 [74.5%] vs. 27 [28.4%]; *p* < 0.001) (Table 2).

**Table 2:** Comparison of clinicopathological characteristics between patients with pN0 and pN1 among patients with cN0

Characteristics	OND (−)	OND (+)	*p*
	*n* = 95	*n* = 47	
Age, yr, median (IQR)	68 [58, 74]	70 [63, 77]	0.159
Sex, male	34 (35.8)	21 (44.7)	0.401
ASA classification, > 2	45 (47.4)	29 (61.7)	0.153
Geographic region, Western countries	75 (78.9)	41 (87.2)	0.332
Incidental diagnosis	75 (78.9)	41 (87.2)	0.335
Cholecystitis	26 (27.4)	11 (23.4)	0.762
Jaundice	8 (8.4)	12 (25.5)	0.012
CA19-9	11.0 [7.0, 25.0]	70.0 [10.0, 1849.0]	< 0.001
SII, median (IQR)	594.3 [426.1, 950.6]	800.3 [483.7, 1462.8]	0.03
PET-CT for preoperative LN assessment	17 (17.9)	5 (10.6)	0.473
*Type of surgery*			0.191
Extended cholecystectomy	42 (44.2)	18 (38.3)	
Cholecystectomy + S4b-S5 resection	51 (53.7)	25 (53.2)	
Cholecystectomy + other liver resection	2 (2.1)	4 (8.5)	
TLNE, median (IQR)	5 [3, 8]	7 [3, 13]	0.023
*Pathological T category*			
T1	18 (18.9)	0 (0.0)	< 0.001
T2	58 (61.1)	24 (51.1)	
T3	17 (17.9)	21 (44.7)	
T4	2 (2.1)	2 (4.3)	
*Pathological N category*			< 0.001
N0	95 (100.0)	0 (0.0)	
N1	0 (0.0)	41 (87.2)	
N2	0 (0.0)	6 (12.8)	
Surgical margin, R0	89 (93.7)	31 (66.0)	< 0.001
Microvascular invasion	41 (43.2)	34 (72.3)	0.002
Grade, poor/undifferentiated	21 (22.1)	12 (25.5)	0.807
Perineural invasion	36 (37.9)	31 (66.0)	0.003
Severe complication	11 (11.6)	7 (14.9)	0.771
Adjuvant chemotherapy	27 (28.4)	35 (74.5)	< 0.001

Values are numbers with percentages in parentheses unless otherwise indicated

*IQR*, interquartile range; *OND*, occult nodal disease; *ASA*, American Society of Anesthesiologists; *CA19-9*, carbohydrate antigen 19-9; *SII*, systemic immune-inflammation index; *LN*, lymph node; *PET-CT*, positron emission tomography-CT; *TLNE*, total number of lymph nodes examined

**Fig. 2:**
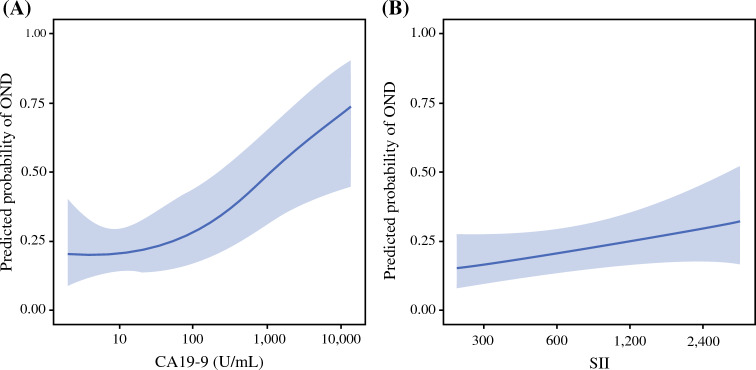
Adjusted restricted cubic spline analyses of carbohydrate antigen 19-9 (CA19-9) (**A**) and systemic immune-inflammation index (SII) (**B**) for predicting occult nodal disease (OND)

On multivariable logistic regression analysis, after adjusting for relevant preoperative and operative variables, preoperative jaundice (OR 3.68, 95% CI 1.21–11.76, *p* = 0.023), higher ln(CA19-9) (OR 1.3, 95% CI 1.12–1.53, *p* = 0.001), higher ln(SII) (OR 1.69, 95% CI 1.05–2.86, *p* = 0.039), and TLNE (OR 1.13, 95%CI 1.05–1.24, *p* = 0.004) were each independently associated with OND (Table 3). Adjusted restricted cubic spline analyses are depicted in Fig. 2. The adjusted predicted probability of OND increased most steeply as CA19-9 rose from approximately 10 to 100 U/mL, followed by a continued upward trend at higher CA19-9 values (Fig. 2A). In contrast, SII exhibited a monotonic, approximately linear association with OND across the observed range (Fig. 2B).

**Table 3:** Univariable and multivariable logistic regression analysis for occult nodal disease among patients with cN0

	Univariable analysis		Multivariable analysis	
Variables	OR 95% CI	*p*	OR 95% CI	*p*
Age	1.03 [0.99, 1.06]	0.111		
Sex, male (ref: female)	1.45 [0.71, 2.96]	0.307		
ASA classification, > 2 (ref: Classification 1,2)	1.79 [0.88, 3.70]	0.109		
Geographic region, Eastern countries (ref: Western countries)	0.55 [0.19, 1.40]	0.234		
Incidental diagnosis	0.66 [0.32, 1.35]	0.252		
Cholecystitis	0.81 [0.35, 1.79]	0.613		
PET-CT for preoperative LN assessment	0.55 [0.17, 1.49]	0.266		
Jaundice	4.31 [1.60, 12.44]	0.005	3.68 [1.21, 11.76]	0.023
Ln(CA19-9)	1.37 [1.18, 1.61]	< 0.001	1.30 [1.12, 1.53]	0.001
Ln(SII)	1.55 [1.01, 2.48]	0.055	1.69 [1.05, 2.86]	0.039
Cholecystectomy + liver resection (ref: Extended cholecystectomy)	1.28 [0.63, 2.64]	0.502		
TLNE	1.11 [1.03, 1.20]	0.006	1.13 [1.05, 1.24]	0.004

*ASA*, American Society of Anesthesiologists; *PET-CT*, positron emission tomography-computed tomography; *LN*, lymph node; *CA19-9*, carbohydrate antigen 19-9; *SII*, systemic immune-inflammation index; *TLNE*, total number of lymph nodes examined

**Fig. 3:**
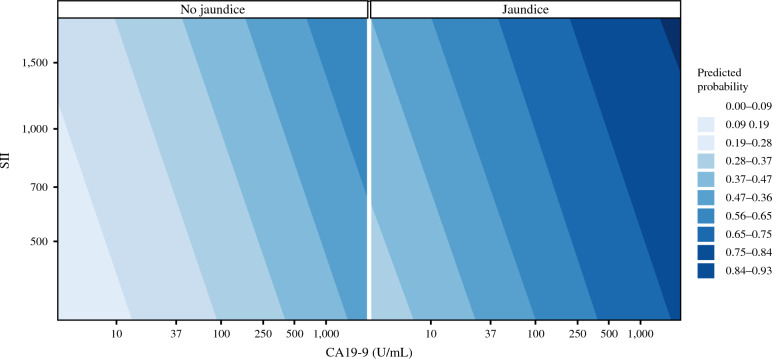
Contour plots of predicted probability of occult nodal disease (OND) according to preoperative carbohydrate antigen 19-9 (CA19-9) and systemic immune-inflammation index (SII), stratified by preoperative jaundice

To further illustrate the joint association of CA19-9 and SII, contour plots of model-predicted probabilities were generated and stratified by preoperative jaundice (Fig. 3). Across the entire range of CA19-9 and SII, the presence of jaundice shifted the predicted probability of OND upward, and higher values of both biomarkers were associated with progressively higher predicted risk, reaching the highest probability bands in patients with concomitantly elevated CA19-9 and SII. Model discrimination was good, with an AUC of 0.73; bootstrap internal validation yielded an optimism-corrected AUC of 0.72.

### Risk Stratification using Preoperative High-risk Markers

**Fig. 4:**
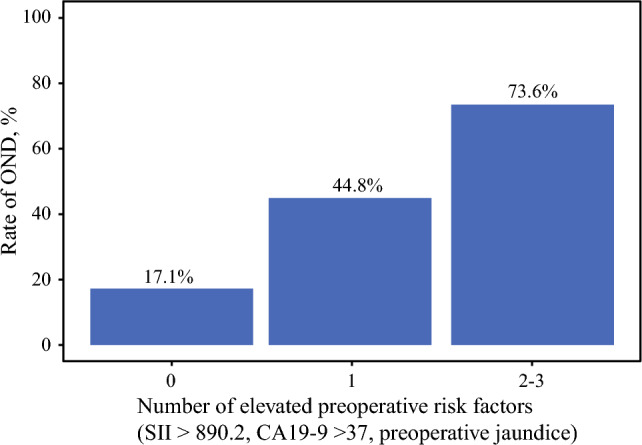
Occult nodal disease (OND) rate according to the number of elevated preoperative risk markers among clinically node-negative (cN0) patients. Markers included high SII (>890.2), CA19-9 (>37 U/mL), and preoperative jaundice

A simple count-based risk stratification using three preoperative markers (high SII > 890.2, CA19-9 > 37 U/mL, and preoperative jaundice) was developed. As noted in Fig. 4, the incidence of OND increased stepwise with the number of elevated markers: 17.1% among patients with 0 markers, 44.8% among individuals with 1 marker, and 73.6% among patients with 2–3 markers.

## Discussion

Although curative-intent resection with regional lymphadenectomy remains the cornerstone of treatment for GBC, outcomes remain poor once LNM is present with reported median OS of only 9.6–15 months.^24^ In the modern era, as systemic therapy options and multimodality strategies have evolved, there is growing interest in treatment intensification and sequencing—such as consideration of neoadjuvant systemic therapy—for selected patients at high risk of recurrence, such as patients with nodal disease, rather than routine upfront surgery.^24–26^ A major challenge, however, is the limited accuracy of preoperative nodal staging in GBC, such that a clinically meaningful subset of patients presumed to be node-negative ultimately harbor nodal metastasis on final pathology.^14–16^ In this context, the current study was important, because it quantified the burden of OND, defined as pathological node-positive disease among clinical node-negative patients, in a contemporary international cohort and identified pragmatic preoperative factors that enriched for OND risk. Notably, OND was present in one-third of patients classified as clinically node-negative. Moreover, preoperative jaundice, CA19-9, and SII were independently associated with OND, and the cumulative burden of these markers provided clear risk separation; OND occurred in more than 70% of patients with two to three elevated markers. Together, these findings support an OND-focused risk enrichment strategy using readily available preoperative data to complement routine staging and inform multidisciplinary decision-making.

With respect to tumor-related markers, both CA19-9 and preoperative jaundice emerged as key predictors of OND. CA19-9 is a widely used tumor-associated biomarker in BTCs and is often considered a surrogate of tumor burden and biologic aggressiveness.^24^ In GBC, elevated CA19-9 has been associated with more advanced disease and a higher likelihood of nodal involvement.^25^ For example, Wang et al. reported that patients with LNM had higher preoperative CA19-9 levels than individuals without nodal metastasis (median, 273.4 U/mL vs. 122.2 U/mL; *p* < 0.01).^26^ Consistent with these observations, CA19-9 remained independently associated with OND after multivariable adjustment in the current cohort, and restricted cubic spline modeling demonstrated a nonlinear association with a steeper rise in OND risk across intermediate CA19-9 values. Preoperative jaundice was also independently associated with OND as patients presenting with jaundice had 3.68-fold higher odds of OND compared with individuals without jaundice. Clinically, jaundice may reflect more locally advanced disease (e.g., biliary obstruction related to tumor extent) and has been associated with inferior outcomes after resection.^27^ Indeed, a meta-analysis demonstrated shorter OS among patients who presented with jaundice (hazard ratio [HR] 2.21; 95% CI 1.64–2.97; *p* < 0.001).^28^ In addition, recent practice frameworks have highlighted biliary obstruction patterns (e.g., perihilar block) as features that may signal anatomically advanced disease and prompt intensified multidisciplinary evaluation.^9^ Taken together, these data suggest that elevated CA19-9 and preoperative jaundice may reflect more aggressive tumor biology and support the potential role of these readily available markers for OND risk enrichment in GBC.

Systemic inflammation has increasingly been recognized as an important dimension of cancer behavior, reflecting host–tumor interactions that may influence invasion and dissemination.^29–31^ In this setting, blood-based inflammatory indices have been investigated as pragmatic prognostic and predictive biomarkers across malignancies.^32–34^ SII, derived from neutrophil, lymphocyte, and platelet counts, integrates neutrophilia and thrombocytosis with relative lymphopenia—features that may collectively capture a protumor inflammatory state and reduced antitumor immune surveillance.^34^ Consistent with this concept, multiple studies have reported that elevated SII is associated with worse survival among patients with GBC, supporting its clinical relevance as a biomarker in this disease.^35,36^ More recently, reports in other tumor types, including intrahepatic cholangiocarcinoma, lung cancer, and endometrial cancer, have linked higher SII with LNM, suggesting a potential role for SII in identifying patients at risk for occult nodal involvement.^37–39^ Against this background, the current study extends prior work by demonstrating that preoperative SII was independently associated with OND among patients staged as clinically node-negative, supporting SII as a practical component of OND risk identification in GBC.

In the current era of evolving multimodality strategies, accurate preoperative identification of nodal disease is increasingly important given the strong adverse prognostic impact of LNM.^9,40^ However, in the current cohort, conventional imaging-based nodal assessment demonstrated limited sensitivity (39.7%) despite high specificity (87.2%), with a positive predictive value of 68.9% and a negative predictive value of 66.9%. These findings underscore a key limitation of contemporary preoperative staging—namely, that a substantial proportion of patients with pathologic nodal metastasis may not be identified before surgery. Indeed, one-third of patients classified as clinically node-negative harbored OND, highlighting a clinically meaningful staging gap. Importantly, the preoperative risk markers identified in this study provided actionable risk enrichment: OND approached approximately 50% when any one of the three markers was present and exceeded 70% when two or more markers were elevated. Taken together, these data suggest that clinically node-negative patients with one or more high-risk markers may warrant intensified preoperative evaluation beyond routine imaging-based staging. One potential adjunct is endoscopic ultrasound (EUS) with fine-needle aspiration (FNA) for targeted sampling of suspicious lymph nodes.^41,42^ In a retrospective cohort of 71 patients with BTCs who underwent EUS-FNA for suspected nodal disease, adequate cytology was obtained in 98.5% with no major complications, and EUS-FNA demonstrated 93% sensitivity and 85% specificity (PPV 87%, NPV 92%, overall accuracy 89%).^43^ In this context, incorporating simple preoperative risk markers (CA19-9, jaundice, and SII) into multidisciplinary decision-making could support several practical, workflow-oriented steps in current practice. Specifically, among clinically node-negative patients with one or more elevated markers, teams may consider intensified regional nodal evaluation using EUS-based assessment with nodal sampling even when CT/MRI/PET-CT suggests cN0. If nodal metastasis is pathologically confirmed by EUS-FNA, these findings may help inform multidisciplinary discussion regarding treatment sequencing, including consideration of systemic therapy prior to surgery in selected patients, while acknowledging that indications for neoadjuvant therapy in GBC remain an evolving area and should be regarded as hypothesis-generating.^9^

Several limitations should be considered when interpreting these findings. The retrospective design introduces the potential for selection bias and residual confounding despite multivariable adjustment. Although the multi-institutional, international nature of the cohort improves generalizability, there was likely heterogeneity in preoperative evaluation, imaging protocols and interpretation, operative technique, and perioperative management across centers, which may have influenced both nodal staging and outcomes. In the present study, TLNE remained independently associated with OND in multivariable analysis, suggesting that the ascertainment of OND may be influenced not only by tumor biology but also by the extent of lymphadenectomy (nodal yield). These findings further support that adequate regional lymphadenectomy is crucial to achieve accurate nodal staging in GBC. In this study, TLNE varied across participating institutions (Supplementary Fig. 1). Moreover, given the retrospective multi-institutional design, the extent of lymphadenectomy—including the nodal stations sampled—and pathology processing protocols were not standardized across centers and instead followed institution-specific protocols, which may have introduced inter-institutional heterogeneity in OND ascertainment. Future prospective studies with standardized minimum nodal yield thresholds, defined nodal station templates, and harmonized pathology protocols are warranted to better delineate the relative contributions of surgical and biological factors to nodal upstaging and to validate preoperative risk stratification strategies. In addition, the proposed marker-based risk enrichment strategy was internally assessed within this cohort and requires external validation and prospective evaluation to confirm reproducibility and clinical utility, including whether it improves decision-making regarding additional staging (e.g., EUS-guided nodal sampling) or treatment sequencing.

## Conclusions

Approximately one in three patients who underwent lymphadenectomy for clinically node-negative gallbladder cancer harbored OND. Preoperative jaundice, elevated CA19-9, and elevated SII were independently associated with OND, and more than 70% of patients with two or more risk markers had OND. Given the limited sensitivity of contemporary imaging for detecting nodal metastasis, integrating these readily available preoperative markers into risk assessment may complement routine staging, help to identify clinically node-negative patients who may warrant additional diagnostic evaluation (e.g., EUS-guided nodal sampling), and inform treatment selection and sequencing, including consideration of neoadjuvant systemic therapy in appropriately selected patients.

## Supplementary Information

Below is the link to the electronic supplementary material.

Supplementary file1 (DOCX 46 KB)

## Data Availability

Study data are not publicly available as they contain patient-level personal information but are available from the corresponding author on reasonable request.
