# Supplementary material for: Occult Nodal Disease in Gallbladder Cancer: An International Multi-institutional Analysis and Preoperative Risk Stratification
Source: Ann Surg Oncol. 2026 Mar 17;33(6):5816–25. doi: 10.1245/s10434-026-19460-0 (PMC13179208; doi:10.1245/s10434-026-19460-0)

**Supplementary Figure 1**: The distribution of the total number of LNs examined (TLNE) across participating institutions


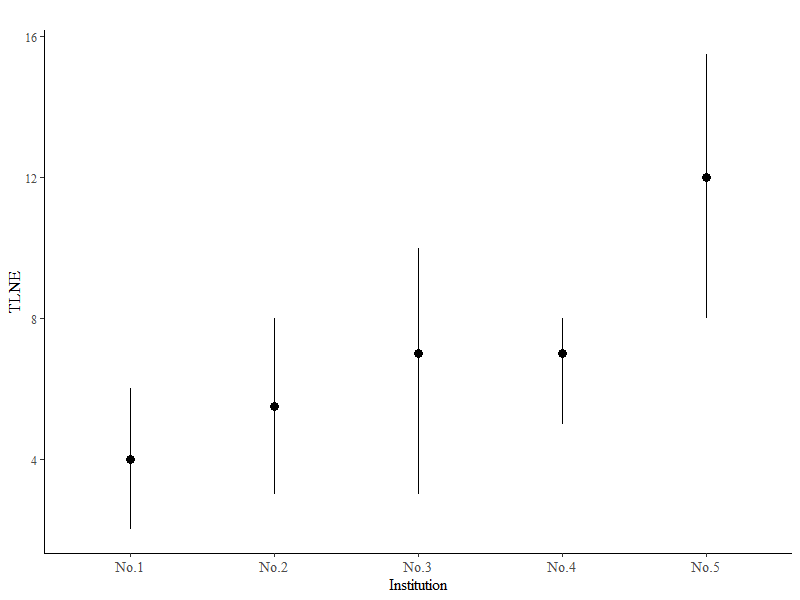

Supplement: Supplementary file 1 — Supplementary file1 (DOCX 46 KB) [file 10434_2026_19460_MOESM1_ESM.docx]
